# Supplementary material for: Mitochondrial DNA editing in mice with DddA-TALE fusion deaminases
Source: Nat Commun. 2021 Feb 19;12:1190. doi: 10.1038/s41467-021-21464-1 (PMC7895935; doi:10.1038/s41467-021-21464-1)
Supplement: Supplementary file 1 — Supplementary Information [file 41467_2021_21464_MOESM1_ESM.docx]

**Supplementary Information**

**Mitochondrial DNA editing in mice with DddA-TALE fusion deaminases**

**Table of Contents**

**Supplementary Figure**

Supplementary Figure 1. Scheme of Golden-Gate cloning for generating the DdCBE construct.

Supplementary Figure 2. ND5 mutant mice (F_0_).

**Supplementary Table**

Supplementary Table 1. TALE arrays used in this study.

**Supplementary Sequences**

Supplementary list of sequences 1. Primers for DdCBE cloning.

Supplementary list of sequences 2. Sequences encoding DdCBE components.

Supplementary list of sequence 3. List of primers used for targeted deep sequencing.


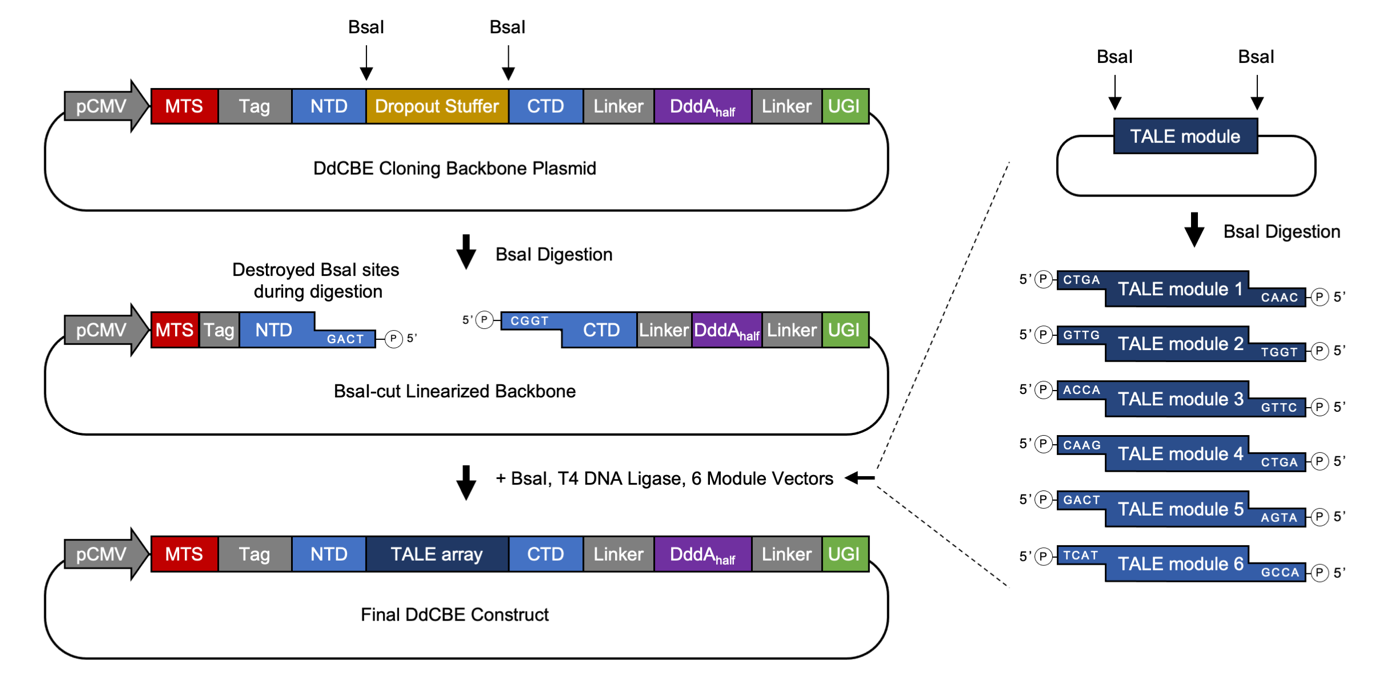


**Supplementary Figure 1. Scheme of Golden-Gate cloning for generating the DdCBE construct.** All reactions simultaneously occur in a single tube; the arrows do not indicate a sequential reaction process. The BsaI enzyme was used to cut the empty expression vector and the module vector, leaving the linearized backbone and TALE module inserts with compatible cohesive end. T4 DNA ligase then ligated the backbone and six module inserts to create the final DdCBE construct. Eight DdCBE cloning backbone plasmids were used in this study: Left-G1333-N, Left-G1333-C, Left-G1397-N, and Left-G1397-C for SOD2 MTS; Right-G1333-N, Right-G1333-C, Right-G1397-N, and Right-G1397-C for COX8A MTS.

**
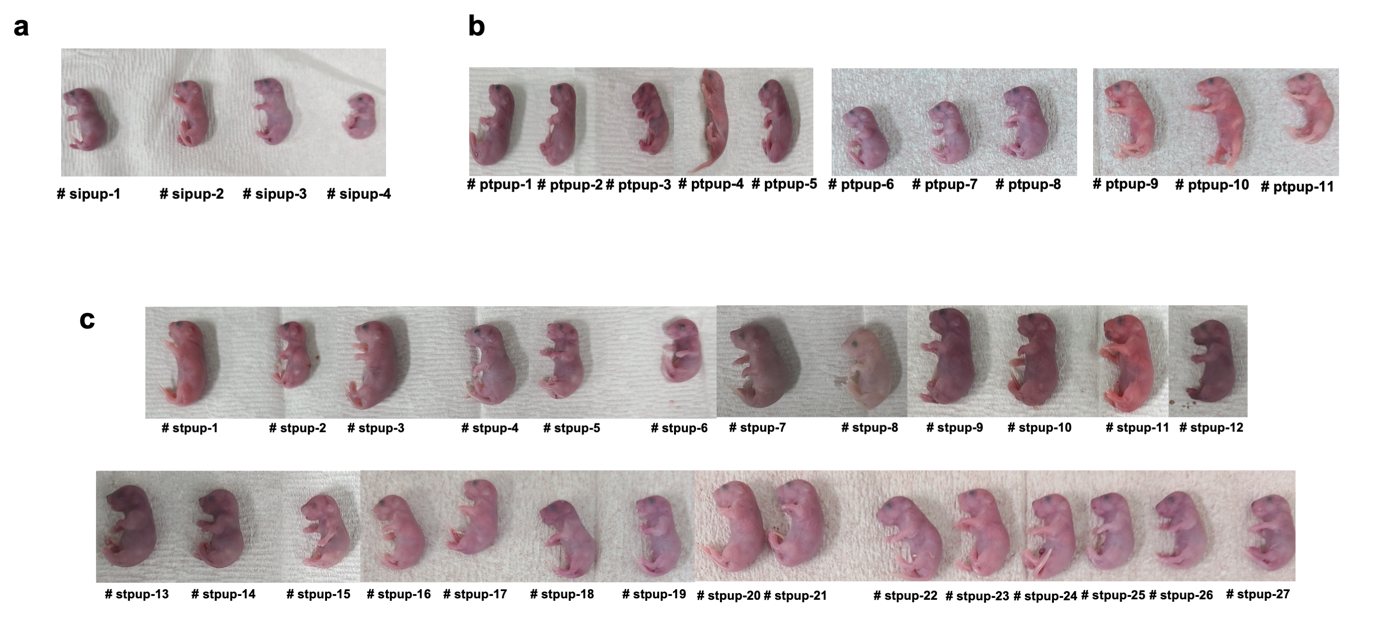
**

**Supplementary Figure 2. ND5 mutant mice (F0).** (a) ND5 silent mutant pups, (b) ND5 G12918A mutant pups, and (c) ND5 nonsense mutant pups, which developed after microinjection of DdCBE mRNAs.

**Supplementary Table 1. TALE arrays used in this study.**

|  | Left TALE  Target Sequence | Right TALE  Target Sequence |
| --- | --- | --- |
| ND5 Silent Muation | 5’-T TTTCCTACTGGTCCGAT T-3’ | 5’-T TAAAATAAAGTTATTAT T-3’ |
| ND5 G12918A Mutation | 5’-T TGCAGGTATTAATTGCT T-3’ | 5’-T TCCTAACAGGGTTCTAC T-3’ |
| ND5 Nonsense Mutation | 5’-T TCCCTAAACATAAACTCA T-3’ | 5’-T TGTTGTTGGAGAATA T-3’ |

We chose TALE arrays with effective DNA binding according to results in our previous report (Kim *et al.*, *Nat. Biotech.,* 31.3 (2013): 251-258). The first and last thymine residues are essential for recognition of target base pairs in our system. The designed TALEs can recognize DNA sequences that are 17–20 bps in length, including a conserved T at the 5′ end.

**Supplementary list of sequences 1. Primers for DdCBE cloning.**

We designed primer sequences that lacked BsaI sites to avoid cleavage of these sequences during Golden-Gate cloning.

Left-MTS-F

ATGGCTCTTAGTCGAGCCGTCTGTGGTACTTCCCGACAGCTTGCTCCAGTCTTGGGGTACCTTGGCTCCCGACAAAAGCACTCTCTCCC

Left-MTS-R

TTGAATTCGGATCCCAGCATAATCAGGGACATCGTATG

Right-MTS-F

ACTCACTATAGGGAGATGGCCTCTGTCTTGACAC

Right-MTS-R

TTGAATTCGGATCCCCTTATCATCATCATCCTTATAATCTATGTCG

Left-NSC-F

GTCCCTGATTATGCTGGGATCCGAATTCAAGATCTACG

Right-NSC-F

GATGATGATGATAAGGGGATCCGAATTCAAGATCTACG

Cterm-R

CATCCAGGGCAGGTCGTCCGCCAAGACAAGCAAGAGCAACAAGATGATCATTTGTCAACGCGGCTAGCGC

Cterm-1333N-F

CTTGTCTTGGCGGACGACCTGCCCTGGATGCTGTGAAAAAGGGGTTGGGGGGCTCTGGCTCTGGTTCCTACGCC

Cterm-1333C-F

CTTGTCTTGGCGGACGACCTGCCCTGGATGCTGTGAAAAAGGGGTTGGGGGGCTCTGGCTCACCAACACCTTATCC

Cterm-1397N-F

CTTGTCTTGGCGGACGACCTGCCCTGGATGCTGTGAAAAAGGGGTTGGGGGGCTCTGGATCAGGTAGCTACGCACT

Cterm-1397C-F

CTTGTCTTGGCGGACGACCTGCCCTGGATGCTGTGAAAAAGGGGTTGGGGGGCTCTGGCAGTGCCATACCTGTG

DddA-R

AGTTGTTTGCCAGTTTCTTTTTCTATGATGTCAGACAGATTTG

UGI-F

ATAGAAAAAGAAACTGGCAAACAACTGGTGATACAAG

UGI-R

ATAGGGCCCTGACTACAACATTTTGATTTTGTTCTCCCC

p3-F

AAAATCAAAATGTTGTAGTCAGGGCCCTATTCTATAGTG

p3-Left-R

AGTACCACAGACGGCTCGACTAAGAGCCATCTCCCTATAGTGAGTCGTATTAATTTC

p3-Right-R

CAAGACAGAGGCCATCTCCCTATAGTGAGTCGTATTAATTTC

**Supplementary list of sequences 2. Sequences encoding DdCBE components.^­^**

Left-SOD2 MTS-3×HA-N terminal domain

MALSRAVCGTSRQLAPVLGYLGSRQKHSLPDYPYDVPDYAGYPYDVPDYAGYPYDVPDYAGIRIQDLRTLGYSQQQQEKIKPKVRSTVAQHHEALVGHGFTHAHIVALSQHPAALGTVAVKYQDMIAALPEATHEAIVGVGKQWSGARALEALLTVAGELRGPPLQLDTGQLLKIAKRGGVTAVEAVHAWRNALTGAPL

Right-COX8A MTS-3×FLAG-N terminal domain

MASVLTPLLLRGLTGSARRLPVPRAKIHSLDYKDHDGDYKDHDIDYKDDDDKGIRIQDLRTLGYSQQQQEKIKPKVRSTVAQHHEALVGHGFTHAHIVALSQHPAALGTVAVKYQDMIAALPEATHEAIVGVGKQWSGARALEALLTVAGELRGPPLQLDTGQLLKIAKRGGVTAVEAVHAWRNALTGAPL

C terminal half domain (NG)-G1333-N-UGI

GLTPEQVVAIASNGGGKQALESIVAQLSRPDPALAALTNDHLVALACLGGRPALDAVKKGLGGSGSGSYALGPYQISAPQLPAYNGQTVGTFYYVNDAGGLESKVFSSGGSGGSTNLSDIIEKETGKQLVIQESILMLPEEVEEVIGNKPESDILVHTAYDESTDENVMLLTSDAPEYKPWALVIQDSNGENKIKML

C terminal half domain (NG)-G1333-C-UGI

GLTPEQVVAIASNGGGKQALESIVAQLSRPDPALAALTNDHLVALACLGGRPALDAVKKGLGGSGSPTPYPNYANAGHVEGQSALFMRDNGISEGLVFHNNPEGTCGFCVNMTETLLPENAKMTVVPPEGAIPVKRGATGETKVFTGNSNSPKSPTKGGCSGGSTNLSDIIEKETGKQLVIQESILMLPEEVEEVIGNKPESDILVHTAYDESTDENVMLLTSDAPEYKPWALVIQDSNGENKIKML

C terminal half domain (NG)-G1397-N-UGI

GLTPEQVVAIASNGGGKQALESIVAQLSRPDPALAALTNDHLVALACLGGRPALDAVKKGLGGSGSGSYALGPYQISAPQLPAYNGQTVGTFYYVNDAGGLESKVFSSGGPTPYPNYANAGHVEGQSALFMRDNGISEGLVFHNNPEGTCGFCVNMTETLLPENAKMTVVPPEGSGGSTNLSDIIEKETGKQLVIQESILMLPEEVEEVIGNKPESDILVHTAYDESTDENVMLLTSDAPEYKPWALVIQDSNGENKIKML

C terminal half domain (NG)-G1397-C-UGI

GLTPEQVVAIASNGGGKQALESIVAQLSRPDPALAALTNDHLVALACLGGRPALDAVKKGLGGSGSAIPVKRGATGETKVFTGNSNSPKSPTKGGCSGGSTNLSDIIEKETGKQLVIQESILMLPEEVEEVIGNKPESDILVHTAYDESTDENVMLLTSDAPEYKPWALVIQDSNGENKIKML

ND5 silent mutation Left TALE repeat

NLTPDQVVAIASNGGGKQALETVQRLLPVLCQAHGLTPAQVVAIASNGGGKQALETVQRLLPVLCQAHGLTPEQVVAIASNGGGKQALETVQRLLPVLCQAHGLTPDQVVAIASHDGGKQALETVQRLLPVLCQAHGLTPAQVVAIASHDGGKQALETVQRLLPVLCQDHGLTPAQVVAIASNGGGKQALETVQRLLPVLCQDHGLTPEQVVAIASNIGGKQALETVQRLLPVLCQAHGLTPDQVVAIASHDGGKQALETVQRLLPVLCQAHGLTPDQVVAIASNGGGKQALETVQRLLPVLCQAHGLTPAQVVAIASNNGGKQALETVQRLLPVLCQDHGLTPAQVVAIASNNGGKQALETVQRLLPVLCQDHGLTPDQVVAIASNGGGKQALETVQRLLPVLCQAHGLTPAQVVAIASHDGGKQALETVQRLLPVLCQDHGLTPDQVVAIASHDGGKQALETVQRLLPVLCQAHGLTPEQVVAIASNNGGKQALETVQRLLPVLCQAHGLTPEQVVAIASNIGGKQALETVQRLLPVLCQAHGLTPDQVVAIASNGGGKQALETVQRLLPVLCQD

ND5 silent mutation Right TALE repeat

NLTPEQVVAIASNGGGKQALETVQRLLPVLCQAHGLTPAQVVAIASNIGGKQALETVQRLLPVLCQDHGLTPAQVVAIASNIGGKQALETVQRLLPVLCQAHGLTPDQVVAIASNIGGKQALETVQRLLPVLCQAHGLTPDQVVAIASNIGGKQALETVQRLLPVLCQDHGLTPDQVVAIASNGGGKQALETVQRLLPVLCQAHGLTPDQVVAIASNIGGKQALETVQRLLPVLCQAHGLTPAQVVAIASNIGGKQALETVQRLLPVLCQDHGLTPAQVVAIASNIGGKQALETVQRLLPVLCQAHGLTPAQVVAIASNNGGKQALETVQRLLPVLCQDHGLTPEQVVAIASNGGGKQALETVQRLLPVLCQAHGLTPAQVVAIASNGGGKQALETVQRLLPVLCQDHGLTPAQVVAIASNIGGKQALETVQRLLPVLCQAHGLTPDQVVAIASNGGGKQALETVQRLLPVLCQAHGLTPDQVVAIASNGGGKQALETVQRLLPVLCQAHGLTPEQVVAIASNIGGKQALETVQRLLPVLCQAHGLTPDQVVAIASNGGGKQALETVQRLLPVLCQD

ND5 point mutation Left TALE repeat

NLTPAQVVAIASNGGGKQALETVQRLLPVLCQAHGLTPDQVVAIASNNGGKQALETVQRLLPVLCQAHGLTPAQVVAIASHDGGKQALETVQRLLPVLCQAHGLTPAQVVAIASNIGGKQALETVQRLLPVLCQAHGLTPAQVVAIASNNGGKQALETVQRLLPVLCQDHGLTPAQVVAIASNNGGKQALETVQRLLPVLCQAHGLTPDQVVAIASNGGGKQALETVQRLLPVLCQAHGLTPDQVVAIASNIGGKQALETVQRLLPVLCQDHGLTPDQVVAIASNGGGKQALETVQRLLPVLCQAHGLTPEQVVAIASNGGGKQALETVQRLLPVLCQAHGLTPAQVVAIASNIGGKQALETVQRLLPVLCQDHGLTPAQVVAIASNIGGKQALETVQRLLPVLCQAHGLTPEQVVAIASNGGGKQALETVQRLLPVLCQAHGLTPAQVVAIASNGGGKQALETVQRLLPVLCQAHGLTPDQVVAIASNNGGKQALETVQRLLPVLCQDHGLTPDQVVAIASHDGGKQALETVQRLLPVLCQAHGLTPAQVVAIASNGGGKQALETVQRLLPVLCQD

ND5 point mutation Right TALE repeat

NLTPAQVVAIASNGGGKQALETVQRLLPVLCQDHGLTPAQVVAIASHDGGKQALETVQRLLPVLCQAHGLTPEQVVAIASHDGGKQALETVQRLLPVLCQAHGLTPEQVVAIASNGGGKQALETVQRLLPVLCQAHGLTPAQVVAIASNIGGKQALETVQRLLPVLCQDHGLTPAQVVAIASNIGGKQALETVQRLLPVLCQAHGLTPEQVVAIASHDGGKQALETVQRLLPVLCQAHGLTPAQVVAIASNIGGKQALETVQRLLPVLCQAHGLTPAQVVAIASNNGGKQALETVQRLLPVLCQAHGLTPAQVVAIASNNGGKQALETVQRLLPVLCQDHGLTPAQVVAIASNNGGKQALETVQRLLPVLCQDHGLTPDQVVAIASNGGGKQALETVQRLLPVLCQAHGLTPEQVVAIASNGGGKQALETVQRLLPVLCQAHGLTPDQVVAIASHDGGKQALETVQRLLPVLCQDHGLTPDQVVAIASNGGGKQALETVQRLLPVLCQAHGLTPEQVVAIASNIGGKQALETVQRLLPVLCQAHGLTPAQVVAIASHDGGKQALETVQRLLPVLCQD

ND5 nonsense mutation Left TALE repeat

NLTPAQVVAIASNGGGKQALETVQRLLPVLCQDHGLTPAQVVAIASHDGGKQALETVQRLLPVLCQAHGLTPEQVVAIASHDGGKQALETVQRLLPVLCQAHGLTPDQVVAIASHDGGKQALETVQRLLPVLCQAHGLTPDQVVAIASNGGGKQALETVQRLLPVLCQDHGLTPAQVVAIASNIGGKQALETVQRLLPVLCQDHGLTPDQVVAIASNIGGKQALETVQRLLPVLCQAHGLTPDQVVAIASNIGGKQALETVQRLLPVLCQAHGLTPAQVVAIASHDGGKQALETVQRLLPVLCQAHGLTPAQVVAIASNIGGKQALETVQRLLPVLCQDHGLTPAQVVAIASNGGGKQALETVQRLLPVLCQDHGLTPDQVVAIASNIGGKQALETVQRLLPVLCQDHGLTPDQVVAIASNIGGKQALETVQRLLPVLCQAHGLTPDQVVAIASNIGGKQALETVQRLLPVLCQAHGLTPAQVVAIASHDGGKQALETVQRLLPVLCQAHGLTPAQVVAIASNGGGKQALETVQRLLPVLCQDHGLTPEQVVAIASHDGGKQALETVQRLLPVLCQAHGLTPAQVVAIASNIGGKQALETVQRLLPVLCQD

ND5 nonsense mutation Right TALE repeat

NLTPAQVVAIASNGGGKQALETVQRLLPVLCQAHGLTPDQVVAIASNNGGKQALETVQRLLPVLCQAHGLTPAQVVAIASNGGGKQALETVQRLLPVLCQDHGLTPAQVVAIASNGGGKQALETVQRLLPVLCQAHGLTPDQVVAIASNNGGKQALETVQRLLPVLCQAHGLTPAQVVAIASNGGGKQALETVQRLLPVLCQDHGLTPEQVVAIASNGGGKQALETVQRLLPVLCQAHGLTPAQVVAIASNNGGKQALETVQRLLPVLCQDHGLTPDQVVAIASNNGGKQALETVQRLLPVLCQAHGLTPDQVVAIASNIGGKQALETVQRLLPVLCQDHGLTPAQVVAIASNNGGKQALETVQRLLPVLCQDHGLTPAQVVAIASNIGGKQALETVQRLLPVLCQAHGLTPEQVVAIASNIGGKQALETVQRLLPVLCQAHGLTPDQVVAIASNGGGKQALETVQRLLPVLCQDHGLTPDQVVAIASNIGGKQALETVQRLLPVLCQA

**Supplementary list of sequences 3. List of primers used for targeted deep sequencing.** Sequences are shown 5’-3’.

For detecting ND5 m.C12539 and m.G12542

1^st^ Forward: CGCAGCTACAGGAAAATCAGC

1^st^ Reverse: ATGGTATTCCTGTGAGGGCG

2^nd^ Forward: ACACTCTTTCCCTACACGACGCTCTTCCGATCTCCTCCACCCATGACTACCAT

2^nd^ Reverse: GTGACTGGAGTTCAGACGTGTGCTCTTCCGATCTGGGTGAGAGCACAAATAGCTG

For detecting ND5 m.G12918

1^st^ Forward: GCCAACTAGGCCTGATAATAG

1^st^ Reverse: GGCGTTTGATTGGGTTTATG

2^nd^ Forward: ACACTCTTTCCCTACACGACGCTCTTCCGATCTGACGAACAAGACATCCGAAA

2^nd^ Reverse: GTGACTGGAGTTCAGACGTGTGCTCTTCCGATCTGCTGTTATAGAAGTGGCGATTA

For detecting ND5 m.C12336 and m.G12341

1^st^ Forward: CACCTCAGCCAACAACAT

1^st^ Reverse: TTGGGTGAGAGCACAAATAG

2^nd^ Forward: ACACTCTTTCCCTACACGACGCTCTTCCGATCTCCCTACAAGCAATCCTCTATAAC

2^nd^ Reverse: GTGACTGGAGTTCAGACGTGTGCTCTTCCGATCTGAGGCCAAATTGTGCTGA
