# Supplementary material for: Mitochondrial DNA editing in mice with DddA-TALE fusion deaminases
Source: Nat Commun. 2021 Feb 19;12:1190. doi: 10.1038/s41467-021-21464-1 (PMC7895935; doi:10.1038/s41467-021-21464-1)
Supplement: Supplementary file 2 — Reporting Summary [file 41467_2021_21464_MOESM2_ESM.pdf]

nature research

Corresponding author(s): Jin-Soo Kim

Last updated by author(s): Jan 27, 2021

Reporting Summary

Nature Research wishes to improve the reproducibility of the work that we publish. This form provides structure for consistency and transparency in reporting. For further information on Nature Research policies, see our Editorial Policies and the Editorial Policy Checklist.

Statistics

For all statistical analyses, confirm that the following items are present in the figure legend, table legend, main text, or Methods section.

n/a	Confirmed	
[ ]	[x]	The exact sample size (n) for each experimental group/condition, given as a discrete number and unit of measurement
[ ]	[x]	A statement on whether measurements were taken from distinct samples or whether the same sample was measured repeatedly
[ ]	[x]	The statistical test(s) used AND whether they are one- or two-sided<br>*Only common tests should be described solely by name; describe more complex techniques in the Methods section.*
[x]	[ ]	A description of all covariates tested
[x]	[ ]	A description of any assumptions or corrections, such as tests of normality and adjustment for multiple comparisons
[ ]	[x]	A full description of the statistical parameters including central tendency (e.g. means) or other basic estimates (e.g. regression coefficient) AND variation (e.g. standard deviation) or associated estimates of uncertainty (e.g. confidence intervals)
[ ]	[x]	For null hypothesis testing, the test statistic (e.g. F, t, r) with confidence intervals, effect sizes, degrees of freedom and P value noted<br>*Give P values as exact values whenever suitable.*
[x]	[ ]	For Bayesian analysis, information on the choice of priors and Markov chain Monte Carlo settings
[x]	[ ]	For hierarchical and complex designs, identification of the appropriate level for tests and full reporting of outcomes
[x]	[ ]	Estimates of effect sizes (e.g. Cohen's d, Pearson's r), indicating how they were calculated

Our web collection on statistics for biologists contains articles on many of the points above.

Software and code

Policy information about availability of computer code

Data collection	no software was used
Data analysis	High-throughput sequencing data was analyzed using CRISPR RGEN Tools (http://www.rgenome.net/). Microsoft Excel (2019) and Powerpoint (2019) was used for drawing figures, graphs, and tables. Genome alignment, primer design, and cloning design were performed with Geneious (version 2021.0.1) and Snapgene 5.2.3 using NC_005089 genome as a reference. Further details and references and provided in the Methods.

For manuscripts utilizing custom algorithms or software that are central to the research but not yet described in published literature, software must be made available to editors and reviewers. We strongly encourage code deposition in a community repository (e.g. GitHub). See the Nature Research guidelines for submitting code & software for further information.

Data

Policy information about availability of data

All manuscripts must include a data availability statement. This statement should provide the following information, where applicable:

- Accession codes, unique identifiers, or web links for publicly available datasets
- A list of figures that have associated raw data
- A description of any restrictions on data availability

Relevant data supporting the findings of this study will be made available in the published article and its Supplementary Information files, the Sequence Read Archive of the NCBI (BioProject accession codes: PRJNA694733 and PRJNA695094), and from the corresponding authors upon reasonable request.

Field-specific reporting

Please select the one below that is the best fit for your research. If you are not sure, read the appropriate sections before making your selection.

- [x] Life sciences
- [ ] Behavioural & social sciences
- [ ] Ecological, evolutionary & environmental sciences

For a reference copy of the document with all sections, see nature.com/documents/nr-reporting-summary-flat.pdf

Life sciences study design

All studies must disclose on these points even when the disclosure is negative.

Sample size	No statistical methods were used to predetermine sample size for in vitro and in vivo experiment. Sample sizes were chosen based on existing procedures and standards in the field.
Data exclusions	There were no data exclusions.
Replication	All cell samples and tissue samples were evaluated in at least biological independent triplicates (n = 3) for calculation of P-value and results were reliably reproduced under the conditions tested.<br>Blastocyst data were generated single biological replicates, because each blastocyst represents an independent entity.
Randomization	Randomization was not required in this study, because we analyzed all experiments performed.
Blinding	Sample collection and analysis were performed by different researchers, without any description.

Reporting for specific materials, systems and methods

We require information from authors about some types of materials, experimental systems and methods used in many studies. Here, indicate whether each material, system or method listed is relevant to your study. If you are not sure if a list item applies to your research, read the appropriate section before selecting a response.

Materials & experimental systems

n/a	Involved in the study	
x		Antibodies
	x	Eukaryotic cell lines
x		Palaeontology and archaeology
	x	Animals and other organisms
x		Human research participants
x		Clinical data
x		Dual use research of concern

Methods

n/a	Involved in the study	
x		ChIP-seq
x		Flow cytometry
x		MRI-based neuroimaging

Eukaryotic cell lines

Policy information about cell lines

Cell line source(s)	NIH/3T3 (CRL-1658) from ATCC®
Authentication	No cell lines were authenticated.
Mycoplasma contamination	Cells were not tested for mycoplasma contamination.
Commonly misidentified lines (See ICLAC register)	No commonly misidentified lines were used .

Animals and other organisms

Policy information about studies involving animals; ARRIVE guidelines recommended for reporting animal research

Laboratory animals	micro injection of mouse zygote. Mice were maintained in a specific pathogen-free (SPF) facility under a 12 h dark-light cycle and constant temperature (20-26 خ) and humidity maintenance (40-60 %).
Wild animals	No wild animals were used.
Field-collected samples	No field-collected samples were used.

Ethics oversight	Approved by the Institutional Animal Care and Use Committee (IACUC) of Institute for Basic Science

Note that full information on the approval of the study protocol must also be provided in the manuscript.
